# Alcohol Consumption in Midlife and Cognitive Performance Assessed 13 Years Later in the SU.VI.MAX 2 Cohort

**DOI:** 10.1371/journal.pone.0052311

**Published:** 2012-12-19

**Authors:** Emmanuelle Kesse-Guyot, Valentina A. Andreeva, Claude Jeandel, Monique Ferry, Mathilde Touvier, Serge Hercberg, Pilar Galan

**Affiliations:** 1 UMR Inserm U557; Inra U1125; Cnam; Université Paris 13 Sorbonne Paris-Cité, CRNH Ile-de-France, Bobigny, France; 2 Centre de Gérontologie Clinique Antonin Balmes, CHU Montpellier, Université I, Montpellier, France; 3 Département de Santé Publique, Hôpital Avicenne, Bobigny, France; University of Chieti, Italy

## Abstract

**Background:**

Associations between alcohol consumption and cognitive function are discordant and data focusing on midlife exposure are scarce.

**Objective:**

To estimate the association between midlife alcohol consumption and cognitive performance assessed 13 y later while accounting for comorbidities and diet.

**Methods:**

3,088 French middle-aged adults included in the SU.VI.MAX (1994) study with available neuropsychological evaluation 13 y later. Data on alcohol consumption were obtained from repeated 24h dietary records collected in 1994–1996**.** Cognitive performance was assessed in 2007–2009 via a battery of 6 neuropsychological tests. A composite score was built as the mean of the standardized individual test scores (mean = 50, SD = 10). ANCOVA were performed to estimate mean differences in cognitive performance and 95% confidence intervals (CI).

**Results:**

In women, abstainers displayed lower cognitive scores than did low-to-moderate alcohol drinkers (1 to 2 drinks/day) (mean difference = −1.77; 95% CI: −3.29, −0.25). In men, heavy drinkers (>3 drinks/day) had higher cognitive scores than did low-to-moderate (1 to 3 drinks/day) (mean difference = 1.05; 95% CI: 0.10, 1.99). However, a lower composite cognitive score was detected in male drinkers consuming ≥90 g/d (≈8 drinks/d). A higher proportion of alcohol intake from beer was also associated with lower cognitive scores. These associations remained significant after adjustment for diet, comorbidities and sociodemographic factors.

**Conclusion:**

In men, heavy but not extreme drinking was associated with higher global cognitive scores. Given the known harmful effects of alcohol even in low doses regarding risk of cancer, the study does not provide a basis for modifying current public health messages.

**Trial Registration:**

ClinicalTrials.gov NCT00272428

## Introduction

At present, treatments to cure or slow the progression of cognitive decline and dementia are not available, arguing for a focus on prevention through health behaviors including physical, social, and cognitive activities, and dietary intake [1]–[3]. Among these modifiable factors, alcohol consumption is of major concern because it is a well-known risk factor for many chronic diseases including fetal alcohol syndrome, neuropsychiatric disorders, cancer, and also for certain risk behaviors (eg, driving under the influence) and injuries [4]–[6]. A more complex relationship has been reported between level of alcohol consumption and cardiovascular disease, as light-to-moderate drinking may in fact display beneficial effects [4], [6].

The association between cognitive function or risk of dementia and alcohol consumption has received a great deal of attention over the past few decades and several reviews of scientific evidence have recently become available [7]–[9]. In particular, the importance of evaluating the sex-specific associations has been highlighted because of fundamental differences between men and women in alcohol consumption patterns and their impact on health/functioning [9] .

The most consistent findings across sex and age concern a somewhat higher risk of cognitive impairment or dementia among abstainers compared to low-to-moderate drinkers [7]. In turn, firm conclusions are not yet established, in particular regarding cognitive decline, because of heterogeneous methodology and relatively low overall quality of the evidence [10]. For example, it has been emphasized that sex-specific and alcoholic beverage-specific data are insufficient, although a beneficial impact of wine intake has been suggested in a few studies [7], [9].

Dietary intake, assessed via holistic approaches, has been associated with cognitive outcomes [11]–[14] and epidemiologic findings suggest a positive correlation between alcohol intake and unhealthy dietary patterns (characterized by increased consumption of meat/processed food and low consumption of plant-derived foods) [15]. In a context where moderate alcohol consumption may help to preserve cognitive function, it is of major interest to assess whether diet indeed accounts for the observed associations. Moderate alcohol consumers have been shown to engage in healthy lifestyles, which has been attributed to their higher educational levels [16].

We hypothesized that midlife alcohol intake (total and by source) would be longitudinally associated with cognitive performance and that these associations would be independent of comorbidities, sociodemographic and economic factors and global quality of the diet.

## Materials and Methods

### Population

The SU.VI.MAX study (1994–2002) included a total of 12,741 French adults (7,713 women aged 35–60 and 5,028 men aged 45–60) and was initially designed as an 8-year randomized double-blind, placebo-controlled primary prevention trial to test the potential efficacy of daily supplementation with antioxidants at nutritional doses on the incidence of cancer, ischemic heart disease and overall mortality [17], [18]. During that period, participants were invited to a yearly check-up consisting of blood sampling alternating with a clinical examination. At the end of the supplementation period (2002), a total of 6,850 subjects had agreed to participate in a post-supplementation observational follow-up, the SU.VI.MAX 2 study [19]. These participants attended a clinical examination during 2007–2009. The SU.VI.MAX and SU.VI.MAX 2 studies were conducted according to the guidelines laid down in the Declaration of Helsinki and were approved by the Ethics Committee for Studies with Human Subjects of Paris-Cochin Hospital (CCPPRB n° 706 and n° 2364, respectively) and the Comité National Informatique et Liberté (CNIL n° 334641 and n° 907094, respectively). Written informed consent was obtained from all subjects.

### Selection Criteria for the Present Study

Among the 6,850 adults included in the SU.VI.MAX 2 study, a total of 4,447 individuals aged 45–60 y at baseline completed all cognitive tests. Among them, 3,362 participants had complete dietary intake data (i.e., at least three complete 24-h dietary records provided during the first two years of follow-up, to account for variability in alcohol intake). From that subsample, we selected a total of 3,088 participants without missing values on any of the covariables for inclusion in the present analysis.

### Alcohol Consumption and Dietary Data

During the SU.VI.MAX study, subjects were invited to complete a 24 h dietary record every 2 months for a total of 6 records per year, randomly assigned across two weekend days and four weekdays per year. Thus, each day of the week and all seasons had an equal chance of being covered. In turn, that allowed taking into account individual variability in intake. Dietary data were collected using the Minitel Telematic Network. The Minitel is a small terminal that was widely used in France as an adjunct to the telephone at the beginning of the SU.VI.MAX study. At enrollment, the participants received a calendar with the assigned days for the 24-hour dietary data reporting and a small central processing unit specifically developed for the study. It contained specialized software that allowed subjects to fill out the computerized dietary record off-line and to transmit the data during brief telephone connections. Participants were assisted by an instruction manual for coding food portions, including validated photographs of more than 250 foods represented in three main portion sizes. Two intermediate and two extreme portions made up a total of seven different portion sizes [20]. Additional assistance was available by telephone.

Detailed information on alcoholic beverage consumption was provided with the 24-h dietary records. Participants could specify intake from a total of 46 different beverage items, including several types of wine, beer, cider and spirits. Accurate quantities were estimated through a set of 30 validated photographs showing various serving (glass) sizes.

Food, alcoholic beverage, and nutrient intakes (including alcohol) were based on the mean intakes across all selected 24-h records. Global dietary quality was calculated using a modified version of the validated *Programme National Nutrition Santé* - Guidelines Score (PNNS-GS), which estimates adherence to French nutrition recommendations. PNNS-GS computation, including food groupings, serving sizes, scoring, and cut-offs, has been described in detail elsewhere [21]. Scoring and cut-off criteria are shown in Supplemental Table 1. For the present analysis, we used a modified score (mPNNS-GS) after removing the alcohol intake and physical activity components.

**Table 1:** Comparison between included and excluded participants regarding baseline characteristics and neuropsychological test scores, SU.VI.MAX 2 Study, 2007–2009 (N = 6,850).

Variable	Excluded participants		Included participants		*P* a
	mean ± sd or %	n with available data	mean ± sd or %	n with available data	
General characteristics					
Male	49	2495	54	3088	*0.0002*
Age at baseline (years)	51±5	2495	52±5	3088	*<0.0001*
BMI (kg/m^2^)	24.5±3.6	2375	24.3±3.4	3088	*0.09*
Education		2359		3088	*0.02*
*Primary*	23		21		
*Secondary*	40		40		
*University or equivalent*	36		39		
Occupational status		2308		3088	*0.001*
*Unemployed*	7		8		
*Manual labor*	5		6		
*Intermediate professions*	55		51		
*Self-employed, farmer*	5		4		
*Managerial staff*	28		32		
Physical activity		2407		3088	*0.04*
*Irregular*	25		23		
*<1h walking/d or equivalent*	30		30		
*≥1h walking/d or equivalent*	45		47		
Smoking status		2230		3088	*0.34*
*Non-smoker*	51		51		
*Former smoker*	37		39		
*Current smoker*	12		10		
Intervention group (1994–2002)	53	2495	53	3088	*0.95*
mPNNS-GS	6.5±1.6	950	6.6±1.6	3088	*0.32*
Alcohol intake (g/d)	19.5±21.1	1086	20.5±20.9	3088	*0.10*
Alcohol categories (drinks/d)		1086		3088	
*Abstainers*	8.8		7.8		*0.21*
*>0 to 2 for women/3 for men*	66.9		65.5		
*>2 for women/>3 for men*	24.2		26.7		
Number of 24-h dietary records	9.7±3.3	1086	10.1±3.1	3088	*<0.0001*
Energy intake b (Kcal/d)	2133±615	1086	2196±610	3088	*<0.0001*
Memory troubles at baseline (yes/no) c	42	1086	36	3088	
RI-48 cued recall task d	25.8±6.3	1410	26.4±6.0	3088	*<0.0001*
Semantic fluency d	28.9±8.1	1396	29.8±8.2	3088	*<0.0001*
Phonemic fluency d	22.1±6.8	1401	22.9±6.6	3088	*<0.0001*
Delis-Kaplan trail-making test e	97.5±42.2	1403	91.4±37.7	3088	*<0.0001*
Forward digit span f	6.8±2.0	1439	7.1±2.0	3088	*<0.0001*
Backward digit span f	6.1±2.1	1438	6.3±2.1	3088	*<0.0001*
CES-D score g	9.8±8.2	2405	8.7±7.4	3088	*<0.0001*

a P values based on Kruskal-Wallis test or chi-squared test.

b Without energy from alcohol.

c Sex-adjusted %.

d Number of words.

e The score on the trail making test is reported as time taken to complete the task. A lower score indicates better performance.

f Number of correct sequences was repeated until the participant failed two consecutive trials of the same digit span.

g CES-D range between 0 and 60; lower score indicates fewer depressive symptoms.

### Cognitive Data

Self-reported memory troubles (yes/no) were recorded at baseline (1994).

During the SU.VI.MAX 2 phase (2007–2009), all participants were invited for a neuropsychological evaluation carried out by trained neuropsychologists. Validated tests limiting floor and ceiling effects were selected as regards cognitive domains affected during brain aging [22], [23]. Episodic memory was evaluated using the RI-48 test, a delayed cued recall test (maximum score of 48) [24]. Lexical-semantic memory was assessed by verbal fluency tasks, including a semantic fluency task (naming as many animals as possible) and a phonemic fluency task (citing words beginning with the letter P). The score was the number of correct words produced during a 2-min period for each task [23]. Working memory was assessed with the forward and backward digit span. One point was scored for each correct sequence repeated, with a maximum score of 14 points for digit span forward as well as backward [25]. Mental flexibility was assessed through the Delis-Kaplan trail-making test (connecting numbers and letters alternating between the two series). The score was the time in seconds needed to complete the task [26].

### Covariates

At baseline, information on gender, date of birth, smoking status, medication use (antihypertensives, anti-diabetic treatments), occupational category, retirement status, physical activity and education was collected. At the first clinical examination (1995–1996), anthropometric measurements and blood pressure (BP) were assessed. Weight was measured using an electronic scale, with subjects wearing indoor clothing and no shoes. Height was measured under the same conditions with a wall-mounted stadiometer. Systolic and diastolic BP were measured twice during a single visit following a 10-min rest and the two measurements were averaged.

At the SU.VI.MAX 2 phase, retirement status and medication use were self-reported. Depressive symptoms were assessed using the French version of the Center for Epidemiologic Studies Depression Scale (CES-D) [27]. BP was assessed using a semi-automatic device (Digital blood pressure monitor OMROM UA-787; OMRON Corporation, Kyoto, Japan). Systolic and diastolic BP were again measured twice during a single visit following a 10-min rest and the two measurements were averaged. Fasting blood samples were obtained at baseline and at the end of the follow-up and all biochemical measurements were centralized. Fasting blood glucose was measured using an enzymatic method (Advia 1650; Bayer Diagnostics). During the entire follow-up, in case of suspected cardiovascular disease, relevant medical data (clinical, biochemical, histological, radiological reports) were requested from participants, physicians and/or hospitals. All reported cardiovascular events were reviewed and validated by an independent expert committee.

### Statistical Analyses

Individual cognitive test scores were converted into T scores (mean = 50, SD = 10) [28]. Thus, a one-point difference in the test score corresponded to one-tenth of a SD difference. A composite cognitive score defined as the mean of the standardized individual test scores was first computed and then rescaled to SD = 10. Thus, a one-point difference in the test score corresponded to a one-tenth of a SD difference. Daily alcohol intakes - total and by source (wine, beer and spirits) - were computed as the mean values across all 24-h records. Total alcohol intake was converted to the number of alcoholic drinks consumed in one day, considering that 1 drink was equivalent to 11 g of alcohol. Daily alcohol use categories were defined as follows: abstainers (0 drinks/d), low-to-moderate drinkers (1 to 3 drinks/d for men and 1 to 2 drinks/d for women) and heavy drinkers (>3 drinks/d for men and >2 drinks/d for women).

In addition, sex-specific tertiles of percent alcohol from wine were computed. For percent alcohol from spirits and beer, a no-consumer category and two categories according to the median value among consumers were defined. Body mass index (BMI) was calculated as the ratio of weight to squared height (kg/m^2^). History of hypertension was defined as systolic BP ≥140 mm Hg or diastolic BP ≥ 90 mm Hg, or reporting of antihypertensive drug use. History of diabetes mellitus was defined as glucose concentrations ≥7 mmol/L or reporting of antidiabetic drug use. Time-dependent retirement status was computed as follows: retired at baseline, retired during follow-up, not yet retired at the end of follow-up.

Included SU.VI.MAX 2 subjects were compared to those excluded from the present study. Descriptive baseline characteristics by sex are reported as mean (SD) or percentage across alcohol consumption categories. Reported P-values refer to a linear contrast test (after a natural log or square root transformation to improve normality), non-parametric Wilcoxon rank test or to the chi^2^ test, as appropriate. Covariance analyses were used to estimate the difference in mean cognitive scores (95% confidence interval) across alcohol intake categories using low-to-moderate drinkers as reference. P for trend was assessed using linear contrast tests across categories. A quadratic contrast was also calculated.

The first ANCOVA model was adjusted for age at the neuropsychological assessment (y) (Model 1). Model 2 was adjusted for age at the neuropsychological assessment (y), number of 24-h dietary records, education (primary, secondary, university or equivalent), supplementation group (active or placebo), baseline BMI (kg/m^2^), memory troubles (yes/no), occupational category (unemployed, manual workers, intermediate professions, self-employed or farmers, managerial staff), tobacco use status (never, former, current), physical activity (irregular, <1h walking/d or equivalent, ≥1h walking/d or equivalent). The third ANCOVA model was further adjusted for comorbidities (depressive symptoms at cognitive evaluation, history of hypertension (yes/no), history of diabetes (yes/no), history of cardiovascular diseases (yes/no)). The final model (Model 4) further included mPNNS-GS and energy intake without alcohol (Kcal/d). Data imputations were carried out in cases of missing mPNNS-GS values. Models focusing on percent alcohol from wine, beer or spirits were adjusted for total alcohol intake.

A secondary analysis was performed using a moredetailed categorisation of alcohol use, as previously suggested [29]. Specifically, the following categories of daily alcohol intake in g/d were considered: 0, 0.1 to 4.9, 5.0 to 14.9, 15.0 to 29.9, 30.0 to 59.9, and ≥60.0 in women; 0, 0.1 to 4.9, 5.0 to 14.9, 15.0 to 29.9, 30.0 to 59.9, 60.0 to 89.9, and ≥90.0 in men.

Finally, in sensitivity analyses, we used inverse probability weighting to correct the estimates for potential selection bias [30]–[32]. First, a logistic regression model was fitted using baseline covariates to predict the probability of inclusion in the present analysis for each participant. Then, the main analysis was rerun using the inverse of the predicted probabilities as weights. All tests of statistical significance were two-sided and the type I error was set at 5%. Statistical analyses were performed using SAS software (version 9.2, SAS Institute Inc, Cary, NC, USA).

## Results

### Comparison of Included and Excluded Participants

Compared with included subjects, subjects in SU.VI.MAX 2 excluded from the current study were younger, with lower levels of education, less often men and less physically active. Excluded subjects also had fewer 24-h records, worse scores on the CES-D and on the neuropsychological tests, and reported more frequent memory troubles at baseline. No difference in alcohol consumption was detected (Table 1).

### Sample Description

At baseline, men and women were 52.4 (4.6) and 51.5 (4.5) years of age, respectively (p<0.001). Daily median alcohol intake was 28.5 g in men and 11.2 g in women (p<0.001).

Characteristics of the participants are presented by gender across alcohol intake categories (Table 2). In both men and women, increasing alcohol consumption was associated with being a former or current smoker, a higher number of 24-h records, higher energy intake and higher percent energy from lipids, lower percent energy form carbohydrates as well as poorer diet quality, estimated through the mPNNS-GS. In men, increasing alcohol intake was also associated with higher percent energy from proteins. In women, heavy drinkers less frequently displayed high levels of physical activity and were more educated.

**Table 2:** Sample characteristics according to alcohol consumption, by sex^1.^

	Men				Women			
Variable	Abstainers(0 drinks/d)	Low to moderate drinkers(>0–≤3 drinks/d)	Heavy drinkers(>3 drinks/d)	*P^b^*	Abstainers(0 drinks/d)	Low to* moderate drinkers(>0–≤2 drinks/d)	Heavy drinkers(>2 drinks/d)	*P* 2
N	58	1010	589		183	1014	234	
Ethanol intake^3^ (g/d)	0	15	49		0	6	31	
Age at baseline (y)	51.6 (4.1)	52.3 (4.6)	52.8 (4.7)	0.07	51.4 (4.5)	51.6 (4.5)	51.5 (4.2)	0.89
Body mass index (kg/m^2^)	24.8 (3.2)	25.0 (2.9)	25.6 (2.8)	0.05	23.4 (3.7)	23.4 (3.6)	23.2 (3.2)	0.72
Energy intake^4^ (Kcal/d)	2151 (551)	2288 (523)	2358 (498)	0.001	1584 (460)	1779 (429)	1801 (433)	<.0001
Total number of 24-h dietary records	8.1 (3.8)	10.3 (3.1)	10.6 (2.7)	<.0001	7.5 (3.6)	10.3 (3.0)	9.9 (2.9)	<.0001
Number of 24-h dietary records (weekday)	5.2 (2.8)	7.2 (2.6)	7.3 (2.4)	<.0001	5.1 (2.8)	7.2 (2.5)	7.0 (2.5)	<.0001
Number of 24-h dietary records (weekend)	2.9 (1.8)	3.2 (1.7)	3.3 (1.7)	0.03	2.4 (1.6)	3.1 (1.7)	2.9 (1.6)	0.001
mPNNS-GS	6.5 (1.8)	6.5 (1.5)	6.1 (1.6)	0.03	6.7 (1.5)	7.0 (1.5)	6.3 (1.5)	0.002
Carbohydrates^5^	45.3 (7.6)	43.0 (6.2)	40.6 (6.1)	<.0001	42.9 (7.5)	42.2 (5.4)	39.4 (5.8)	<.0001
Lipids^5^	37.6 (5.8)	39.4 (5.1)	41.1 (5.1)	<.0001	38.5 (6.2)	40.1 (4.7)	42.4 (4.7)	<.0001
Protein^5^	17.1 (3.7)	17.6 (2.7)	18.3 (2.6)	<.0001	18.6 (3.7)	17.7 (2.6)	18.2 (2.5)	0.37
Active supplementation group (yes/no, 1994–2002)	60	53	53	0.68	57	53	49	0.08
Self-reported memory troubles (yes/no)	36	27	26	0.28	48	44	44	0.50
Occupational categories				0.44				0.03
*Unemployed*	0	0	0		8	7	9	
*Manual labor*	3	6	7		4	2	3	
*Intermediate professions*	50	42	41		69	69	62	
*Self-employed, farmer*	10	5	6		8	4	3	
*Managerial staff*	36	46	45		11	17	23	
Education				0.32				0.02
*Primary*	19	22	22		25	20	18	
*Secondary (high school)*	34	34	37		42	46	38	
*University or equivalent*	47	44	41		33	34	44	
Physical activity				0.63				0.01
*Irregular*	17	22	24		17	24	25	
*<1h walking/d or equivalent*	28	24	22		34	36	41	
*≥1h walking/d or equivalent*	55	53	54		48	40	34	
Smoking status				<.0001				<.0001
*Never*	43	43	27		71	68	51	
*Former*	41	47	57		24	25	32	
*Current*	16	9	16		5	7	17	

Abbreviations: mPNNS-GS: modified Programme National Nutrition Santé-Guidelines Score.

1 Values are mean (SD) or % as appropriate except when otherwise noted.

2 P is based on linear contrast (log-transformed data except for mPNNS-GS) or chi^2^ test, as appropriate.

3 Median values.

4 Energy intake without alcohol.

5 Percent of energy intake (without alcohol).

### Association between Global Cognitive Function and Midlife Total Alcohol Intake

Associations between the composite cognitive score and alcohol intake are presented in Table 3. In the age-adjusted model (Model 1), heavy drinking was associated with better cognitive performance. The association was slightly attenuated after adjustment for age, number of 24-h records, BMI, baseline memory troubles, education, occupational category, tobacco use status and physical activity (Models 2 and 3). This association was not modified by further adjustment for comorbidities, energy intake and mPNNS-GS (Model 4).

**Table 3:** ANCOVA-derived associations between alcohol intake and global cognitive function, by sex^1.^

		Alcohol intake		
	Abstainers	low to moderate drinkers	heavy drinkers	*P* 2
**Men**				
Model 1^3^	−0.28 (−2.87–2.31)	0 (reference)	**1.06 (0.07–2.06)**	*0.32*
Model 2^4^	0.04 (−2.38–2.47)	0 (reference)	**1.03 (0.09–1.97)**	*0.44*
Model 3^5^	−0.03 (−2.45–2.39)	0 (reference)	**1.04 (0.10–1.98)**	*0.40*
Model 4^6^	0.04 (−2.39–2.46)	0 (reference)	**1.05 (0.10–1.99)**	*0.43*
**Women**				
Model 1^3^	**−2.91 (−4.46–1.36)**	0 (reference)	1.32 (−0.08–2.72)	*<0.0001*
Model 2^4^	**−2.04 (−3.54–0.54)**	0 (reference)	0.71 (−0.61–2.03)	*0.003*
Model 3^5^	**−2.05 (−3.56–0.54)**	0 (reference)	0.76 (−0.55–2.08)	*0.003*
Model 4^6^	**−1.77 (−3.29–0.25)**	0 (reference)	1.03 (−0.31–2.36)	*0.003*

1 Values are mean difference (95% confidence interval) in composite cognitive score, low to moderate drinkers as reference.

2 P for trend across categories.

3 Model 1 is adjusted for age.

4 Model 2 : model 1+ number of 24-h records, BMI, baseline memory troubles, education, occupational category, tobacco use status and physical activity.

5 Model 3: model 2+ supplementation group during the trial phase, depressive symptoms concomitant with cognitive function assessment, history of diabetes, hypertension, or cardiovascular disease.

6 Model 4: model 3+ energy intake and mPNNS-GS.

**Figure 1:**
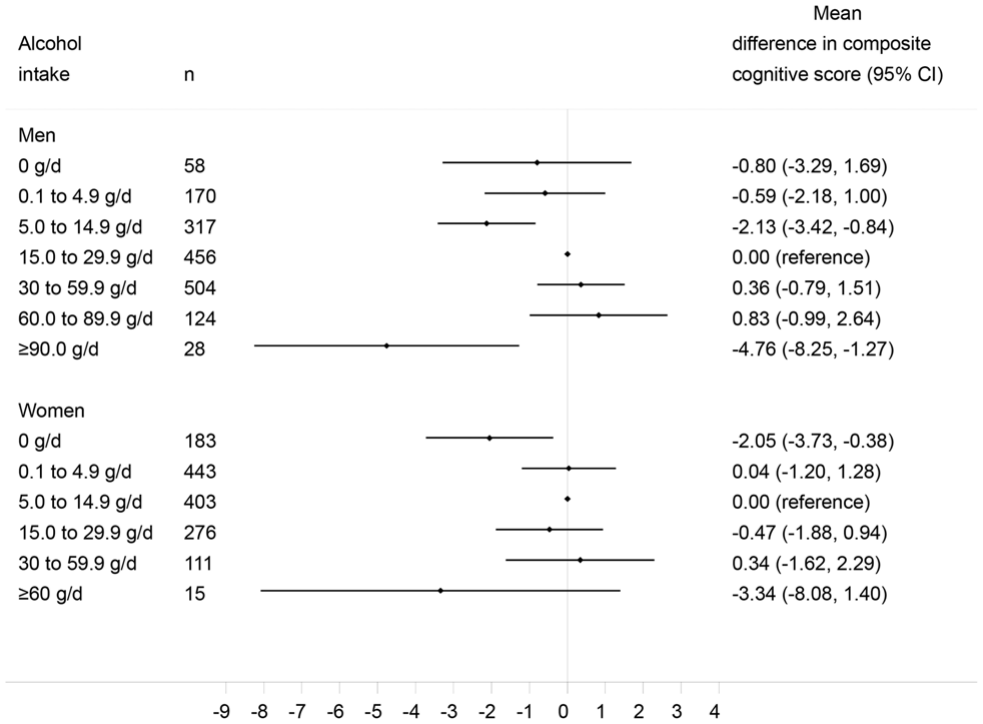
ANCOVA-derived associations between alcohol intake categories and global cognitive function, by sex. Association between the composite cognitive score and alcohol consumption using a detailed categorization of the exposure level. Models adjusted for age, number of 24-h records, BMI, baseline memory troubles, education, occupational category, tobacco use status, physical activity, supplementation group during the trial phase, depressive symptoms concomitant with cognitive function assessment, history of diabetes/hypertension/cardiovascular disease, energy intake and mPNNS-GS. Values are mean difference and 95% confidence interval.

In women, lower cognitive performance scores were detected among abstainers compared to low-to-moderate drinkers in the age-adjusted model. Despite a substantial reduction of the extent of the association after adjustment for age, number of 24-h records, BMI, baseline memory troubles, education, occupational category, tobacco use status, physical activity comorbidities (depressive symptoms, history of diabetes, hypertension, or cardiovascular disease) as well as energy intake and mPNNS-GS (model 4), the lower cognitive performance scores among abstainers remained statistically significant. The findings of the secondary analysis are shown in Figure 1. Despite a loss of power due to the increase in the number of categories, elevated alcohol consumption (≥90 g/d ≈ 8 drinks/d) among men was associated with a lower cognitive performance compared to consumption of 15.0 to 29.9 g/d (≈1.4 to 2.7 drinks/d).

### Association between Global Cognitive Function and Midlife Alcohol Intake by Source

Median percentages of alcohol intake from beer, spirits and wine were 2.45, 6.78, 82.40 and 0.0, 6.18, 78.91 in men and women, respectively. Wine was by far the biggest contributor to alcohol intake. In turn, alcohol intake in terms of percent ethanol from each source, largely provided no significant results regarding cognition after adjustment for total intake of alcohol, except for an inverse association between ethanol intake from beer and cognitive performance in men. In particular, compared to men with low ethanol intake from beer (0 - median value among consumers = 10.4% of ethanol), those with high intake (>10.4% of ethanol) showed poorer cognitive performance (mean difference = −1.27, 95% confidence interval: −2.45, −0.09) (data not tabulated).

### Association between Individual Cognitive Tests and Alcohol Intake

Associations between each individual cognitive test and alcohol intake are presented in Table 4. No significant association was detected among men. Among women, a similar association as the one with the composite cognitive score was observed between alcohol intake and scores on semantic fluency and with the RI-48 cued recall task. On the opposite, compared to low-to-moderate drinkers, women who were heavy drinkers displayed better scores for phonemic fluency.

### Sensitivity Analysis

Results of the additional analyses using inverse probability weighting are presented in Table 5. Estimates and confidence intervals were slightly modified but similar associations were detected.

## Discussion

In this population of French adults, alcohol abstinence was associated with somewhat decreased cognitive performance compared to low-to-moderate alcohol drinking in women. The overall association was largely driven by performance on episodic memory and semantic fluency tasks. In men, heavy drinking (>3 drinks/d) was associated with better cognitive performance, particularly on the backward digit span task. After splitting the alcohol use categories, men with alcohol intake ≥90 g/d (corresponding to about 8 drinks/d) showed lower cognitive performance compared to those consuming ∼ 1.4–2.7 drinks/d. An inverse association between ethanol intake from beer and cognitive performance was also observed.

### Alcohol intake and Cognitive Function

Our findings in women are in accordance with various previous studies investigating the relationship between alcohol intake and cognitive outcomes, and reporting a favorable association of low-to-moderate drinking with subsequent cognitive functioning [7], [9]. The significant association detected only in women is in line with the results of several other studies [33]–[36] showing sex-specific associations. It has generally been speculated that the inconsistent testing of sex-specific models could partly account for the observed discrepancies among studies. Indeed, the very small number of abstainers, particularly among middle-aged men, and of heavy drinkers, especially among women, may have prevented the detection of a potential relationship. Further, heterogeneous findings may have resulted partly as a consequence of omitted confounders of the effects of alcohol use. Among them, diet, comorbidities involving changes in alcohol use habits or social factors could be considered. Indeed, former drinkers, who had possibly discontinued alcohol consumption as a result of vascular diseases, are usually included with abstainers. This may have led to a classification bias resulting in lower cognitive functioning in non-drinkers. A recent study focusing on the association between alcohol intake and cognitive decline did not find a beneficial effect of low-to-moderate drinking compared to abstinence when former drinkers were separately accounted for [37]. In the present study, adjustment for comorbidities occurring during the follow-up did not substantially modify the results.

**Table 4:** Associations between alcohol intake and specific cognitive tests, by sex1,2.

		Alcohol intake		
	abstainers	low to moderate drinkers	heavy drinkers	*P* 2
**Men**				
Backward digit span	1.55 (−1.13–4.22)	0 (reference)	1.04 (−0.01–2.08)	*0.71*
Forward digit span	0.86 (−1.81–3.53)	0 (reference)	0.88 (−0.16–1.92)	*0.99*
Trail-making test	−1.17 (−3.58–1.24)	0 (reference)	−0.05 (−0.99–0.89)	*0.38*
RI-48 cued recall task	−1.24 (−3.83–1.35)	0 (reference)	0.72 (−0.29–1.74)	*0.15*
Semantic fluency	0.61 (−1.87–3.10)	0 (reference)	0.59 (−0.38–1.56)	*0.99*
Phonemic fluency	−0.47 (−3.03–2.09)	0 (reference)	0.86 (−0.14–1.86)	*0.32*
**Women**				
Backward digit span	−0.52 (−2.08–1.03)	0 (reference)	1.00 (−0.37–2.36)	*0.12*
Forward digit span	−0.81 (−2.40–0.78)	0 (reference)	0.41 (−0.98–1.81)	*0.21*
Trail-making test	−0.46 (−2.06–1.14)	0 (reference)	−0.54 (−1.94–0.86)	*0.93*
RI-48 cued recall task	**−2.25 (−3.88–0.62)**	0 (reference)	0.07 (−1.36–1.50)	*0.02*
Semantic fluency	**−1.67 (−3.31–0.03)**	0 (reference)	0.84 (−0.59–2.28)	*0.01*
Phonemic fluency	−1.11 (−2.68–0.47)	0 (reference)	**2.17 (0.79–3.55)**	*0.001*

1 Values are mean difference (95% confidence interval) in composite cognitive score, low to moderate drinkers as reference.

2 Models adjusted for age, number of 24-h records, BMI, baseline memory troubles, education, occupational category, tobacco use status, physical activity, supplementation group during the trial phase, depressive symptoms concomitant with cognitive function assessment, history of diabetes/hypertension/cardiovascular disease, energy intake and mPNNS-GS.

3 P for trend across categories.

Our SU.VI.MAX 2 data showed a slight reduction in the mean difference in women’s cognitive functioning between low-to-moderate drinkers and abstainers after accounting for the quality of diet, but the association remained statistically significant. Indeed, low-to-moderate drinking in women was associated with better adherence to existing nutrition recommendations in our population (data not shown). The positive association between heavy drinking and cognitive function observed in men was not substantially modified after accounting for the quality of the diet, given that the adverse effects of high alcohol intake could have been at least partly due to poor diet quality. While we are not aware of any relevant studies accounting for dietary confounders, epidemiologic data on the association between dietary behaviors and alcohol intake are plentiful [15], [38]. Likewise, a differential impact of alcohol intake on cognitive outcomes according to social position was recently reported [39]. In that study among French blue-collar workers, lower cognitive functioning was observed among heavy drinkers compared to low-to-moderate drinkers, but that association was restricted to men of low socio-economic status. We did not retrieve such an interaction in our population (data not shown).

In our study, the association between alcohol consumption and cognitive function remained even after accounting for major confounders, suggesting a true association which could be corroborated by mechanistic hypotheses. The mechanisms underlying the potential beneficial effect of alcohol intake on brain aging encompass lipoprotein level modification and improvement of cerebral blood flow [40]. Some experimental studies further suggest that ethanol may directly affect brain structure [41], [42]. Indirect effects through the antioxidant and anti-inflammatory properties of flavonoids found in red wine (resveratrol) have also been postulated [42].

**Table 5:** Associations between alcohol intake and cognitive tests, by sex: inverse probability-weighted models1,2.

		Alcohol intake		
	abstainers	low to moderate drinkers	heavy drinkers	*P* 2
**Men**				
Composite cognitive score	0.41 (−1.83–2.66)	0 (reference)	**1.03 (0.08–1.98)**	*0.60*
Backward digit span	1.91 (−0.57–4.39)	0 (reference)	**1.07 (0.03–2.12)**	*0.52*
Forward digit span	1.17 (−1.32–3.65)	0 (reference)	0.83 (−0.22–1.87)	*0.80*
Trail-making test	−1.09 (−3.33–1.15)	0 (reference)	−0.12 (−1.06–0.83)	*0.41*
RI-48 cued recall task	−0.89 (−3.29–1.52)	0 (reference)	0.73 (−0.28–1.75)	*0.20*
Semantic fluency	0.66 (−1.64–2.97)	0 (reference)	0.59 (−0.38–1.56)	*0.95*
Phonemic fluency	−0.16 (−2.54–2.21)	0 (reference)	0.86 (−0.14–1.86)	*0.41*
**Women**				
Composite cognitive score	**−1.81 (−3.26–0.36)**	0 (reference)	0.88 (−0.46–2.22)	*0.003*
Backward digit span	−0.63 (−2.11–0.84)	0 (reference)	0.99 (−0.37–2.35)	*0.08*
Forward digit span	−0.82 (−2.33–0.70)	0 (reference)	0.27 (−1.13–1.66)	*0.26*
Trail-making test	−0.48 (−2.01–1.05)	0 (reference)	−0.73 (−2.15–0.68)	*0.79*
RI-48 cued recall task	**−2.19 (−3.74–0.63)**	0 (reference)	−0.01 (−1.44–1.43)	*0.03*
Semantic fluency	**−1.73 (−3.29–0.17)**	0 (reference)	0.79 (−0.65–2.23)	*0.01*
Phonemic fluency	−1.13 (−2.63–0.38)	0 (reference)	**2.09 (0.71–3.48)**	*0.001*

1 Values are mean difference (95% confidence interval) in composite cognitive score, low to moderate drinkers as reference.

2 Models adjusted for age, number of 24-h records, BMI, baseline memory troubles, education, occupational category, tobacco use status, physical activity, supplementation group during the trial phase, depressive symptoms concomitant with cognitive function assessment, history of diabetes/hypertension/cardiovascular disease, energy intake and mPNNS-GS.

3 P for trend across categories.

Some epidemiologic data support the hypothesis of a harmful effect of heavy drinking on cognitive performance but findings are not consistent [7]. We observed a significant mean difference in cognitive function among heavy drinkers compared to low-to-moderate drinkers only in men. However, heavy drinking is a heterogeneous category including people with various levels of intake. In particular, volunteering for an active follow-up could have led to a selection bias towards including participants who are not “heavy drinkers” by common standards. Despite the low number of such drinkers in the SU.VI.MAX study, the findings of our secondary analysis showing that extreme heavy drinking could have an adverse association with cognitive function suggest that alcohol intake and cognition may exhibit an inverted U or J shape.

In the present study, specific neuropsychological tests, namely those related to episodic memory, semantic fluency and backward digit span were associated with alcohol intake. This is in line with other findings [36], [43]–[46] suggesting that alcohol may differentially affect cognitive domains.

### Type of Alcoholic Beverages and Cognitive Function

In this study, no differences in cognitive function according to the type of beverage consumed were observed. One exception was the finding of lower cognitive performance scores among men with a high contribution of beer to their total alcohol intake. Nevertheless, alcohol intake was mainly provided by wine and thus highly correlated with wine consumption, preventing a clear distinction between specific alcohol sources. Some authors have focused on the association between cognitive outcomes and alcohol intake by source [7]. To the best of our knowledge, the relative contribution of each source to total alcohol intake has not been highlighted, however, authors focusing on the type of alcoholic beverage after accounting for other sources do not report specific effects [47]. Nonetheless, authors have distinguished between “wet” (as in France) and “dry” drinking cultures as regards quantities and patterns of drinking. Future analyses using appropriate designs and comparing associations between alcohol intake and cognitive outcomes across countries may help improve understanding of the independent impact of the particular drinking culture [48].

### Limitations

Some limitations of our study should be noted. First, cognitive evaluation was not available at baseline, thus adjustment for baseline differences in cognitive performance according to midlife alcohol consumption was not possible. Preexisting differences in cognitive performance may have led to differences in alcohol consumption. However, our sample included relatively young and healthy volunteers, arguing for the likely absence of cognitive impairment at baseline. In addition, we were also not able to focus on cognitive decline over time. Second, we were not able to firmly distinguish between abstainers and former drinkers, acknowledging that former drinkers may be an at-risk population as quitters. The impact of that limitation, however, might be small in our study since we used midlife exposure assessment and adjustment for comorbidities. In addition, abstainers were not more likely to be hypertensive and were in fact less likely to have diabetes. Third, as wine was the dominant source of alcohol in our population, we were unable to separate the effects of wine from those of alcohol. Finally, caution is advised when assessing the external validity of our results as we cannot assume that our population is representative of the general population. Indeed, these analyses were based on a subsample of the SU.VI.MAX cohort, whose participants generally had a higher educational level and occupational status, along with a healthier diet than the general French population. In particular, subjects with available cognitive and alcohol data may have been particularly health-conscious, as shown by the comparison between included and excluded participants, limiting the generalizability of the findings. Nonetheless, use of inverse probability weighting to correct for potential selection bias did not modify the findings.

### Strengths

In turn, some strengths of our analysis should also be emphasized. The long follow-up allowed focusing on alcohol intake as a midlife exposure. Further, food and alcoholic beverage consumption was assessed through repeated 24h dietary records strengthening the accuracy of the collected exposure data. Finally, standardized cognitive evaluation was completed in a relatively young population and a neuropsychological battery of sensitive tests was used to limit floor or ceiling effects.

### Conclusion

In conclusion, while providing evidence for sex-specific associations, our results support the hypothesis that midlife low-to-moderate drinking in women and even heavy drinking in men might be associated with better cognitive functioning, independent of diet or comorbidities. However, extreme alcohol consumption may be harmful for cognitive function at least among men. Albeit sex-specific, the definitions of low-to-moderate and heavy drinking are not clear-cut. There is substantial evidence from other health domains (e.g., cancer development) that even a single serving of alcohol might have a detrimental impact.

## References

[1] Middleton LE, Yaffe K (2009) Promising strategies for the prevention of dementia. Arch Neurol 66: 1210–1215. 19822776 10.1001/archneurol.2009.201 PMC2762111

[2] de la Torre JC (2010) Alzheimer’s disease is incurable but preventable. J Alzheimers Dis 20: 861–870. 5W2WM8876N781201 [pii];10.3233/JAD-2010-091579 [doi]. 10.3233/JAD-2010-091579 20182017

[3] Coley N, Andrieu S, Gardette V, Gillette-Guyonnet S, Sanz C, et al (2008) Dementia prevention: methodological explanations for inconsistent results. Epidemiol Rev 30: 35–66. 18779228 10.1093/epirev/mxn010

[4] WHO (2011) Global Status Report on Alcohol and Health 2011. Geneva.

[5] Siliquini R, Bert F, Alonso F, Berchialla P, Colombo A, et al. (2011) Correlation between driving-related skill and alcohol use in young-adults from six European countries: the TEN-D by Night Project. BMC Public Health 11: 526. 1471-2458-11-526 [pii];10.1186/1471-2458-11-526 [doi]. 10.1186/1471-2458-11-526 PMC3145590 21722358

[6] Parry CD, Patra J, Rehm J (2011) Alcohol consumption and non-communicable diseases: epidemiology and policy implications. Addiction 106: 1718–1724. 10.1111/j.1360-0443.2011.03605.x [doi]. 10.1111/j.1360-0443.2011.03605.x PMC3174337 21819471

[7] Neafsey EJ, Collins MA (2011) Moderate alcohol consumption and cognitive risk. Neuropsychiatr Dis Treat 7: 465–484. 10.2147/NDT.S23159 [doi];ndt-7-465 [pii]. 10.2147/NDT.S23159 PMC3157490 21857787

[8] Peters R, Peters J, Warner J, Beckett N, Bulpitt C (2008) Alcohol, dementia and cognitive decline in the elderly: a systematic review. Age Ageing 37: 505–512. afn095 [pii];10.1093/ageing/afn095 [doi]. 10.1093/ageing/afn095 18487267

[9] Panza F, Capurso C, D’Introno A, Colacicco AM, Frisardi V, et al. (2009) Alcohol drinking, cognitive functions in older age, predementia, and dementia syndromes. J Alzheimers Dis 17: 7–31. 38M3600311336263 [pii];10.3233/JAD-2009-1009 [doi]. 10.3233/JAD-2009-1009 19494429

[10] Plassman BL, Williams JW, Burke JR, Holsinger T, Benjamin S (2010) Systematic review: factors associated with risk for and possible prevention of cognitive decline in later life. Ann Intern Med 153: 182–193. 0003-4819-153-3-201008030-00258 [pii];10.1059/0003-4819-153-3-201008030-00258 [doi]. 10.7326/0003-4819-153-3-201008030-00258 20547887

[11] Tangney CC, Kwasny MJ, Li H, Wilson RS, Evans DA, et al. (2011) Adherence to a Mediterranean-type dietary pattern and cognitive decline in a community population. Am J Clin Nutr 93: 601–607. ajcn.110.007369 [pii];10.3945/ajcn.110.007369 [doi]. 10.3945/ajcn.110.007369 PMC3041601 21177796

[12] Gu Y, Scarmeas N (2011) Dietary Patterns in Alzheimer’s Disease and Cognitive Aging. Curr Alzheimer Res. BSP/CAR/0176 [pii]. 10.2174/156720511796391836 PMC3283139 21605048

[13] Kesse-Guyot E, Amieva H, Castetbon K, Henegar A, Ferry M, et al (2011) Adherence to nutritional recommendations and subsequent cognitive performance: findings from the prospective Supplementation with Antioxidant Vitamins and Minerals 2 (SU.VI.MAX 2) study. Am J Clin Nutr 93: 200–210. 21106918 10.3945/ajcn.2010.29761

[14] Gustaw-Rothenberg K (2009) Dietary patterns associated with Alzheimer’s disease: population based study. Int J Environ Res Public Health 6: 1335–1340. 19440521 10.3390/ijerph6041335 PMC2681193

[15] Newby PK, Tucker KL (2004) Empirically derived eating patterns using factor or cluster analysis: a review. Nutr Rev 62: 177–203. 15212319 10.1301/nr.2004.may.177-203

[16] Hansel B, Thomas F, Pannier B, Bean K, Kontush A, et al. (2010) Relationship between alcohol intake, health and social status and cardiovascular risk factors in the Urban Paris-Ile-de-France Cohort: is the cardioprotective action of alcohol a myth? Eur J Clin Nutr 64: 561–568. ejcn201061 [pii];10.1038/ejcn.2010.61 [doi]. 10.1038/ejcn.2010.61 20485310

[17] Hercberg S, Galan P, Preziosi P, Roussel AM, Arnaud J, et al (1998) Background and rationale behind the SU.VI.MAX Study, a prevention trial using nutritional doses of a combination of antioxidant vitamins and minerals to reduce cardiovascular diseases and cancers. SUpplementation en VItamines et Mineraux AntioXydants Study. Int J Vitam Nutr Res 68: 3–20. 9503043

[18] Hercberg S, Galan P, Preziosi P, Bertrais S, Mennen L, et al (2004) The SU.VI.MAX Study: a randomized, placebo-controlled trial of the health effects of antioxidant vitamins and minerals. Arch Intern Med 164: 2335–2342. 15557412 10.1001/archinte.164.21.2335

[19] Kesse-Guyot E, Fezeu L, Jeandel C, Ferry M, Andreeva V, et al. (2011) French adults’ cognitive performance after daily supplementation with antioxidant vitamins and minerals at nutritional doses: a post hoc analysis of the Supplementation in Vitamins and Mineral Antioxidants (SU.VI.MAX) trial. Am J Clin Nutr. ajcn.110.007815 [pii];10.3945/ajcn.110.007815 [doi]. 10.3945/ajcn.110.007815 21775560

[20] Le Moullec N, Deheeger M, Preziosi P, Montero P, Valeix P, et al (1996) Validation du manuel photos utilisé pour l’enquête alimentaire de l’étude SU.VI.MAX. Cahier de Nutrition et de Diététique 31: 158–164.

[21] Estaquio C, Kesse-Guyot E, Deschamps V, Bertrais S, Dauchet L, et al (2009) Adherence to the French Programme National Nutrition Sante Guideline Score is associated with better nutrient intake and nutritional status. J Am Diet Assoc 109: 1031–1041. 19465185 10.1016/j.jada.2009.03.012

[22] Amieva H, Jacqmin-Gadda H, Orgogozo JM, Le CN, Helmer C, et al. (2005) The 9 year cognitive decline before dementia of the Alzheimer type: a prospective population-based study. Brain 128: 1093–1101. awh451 [pii];10.1093/brain/awh451 [doi]. 10.1093/brain/awh451 15774508

[23] Lezak MD, Howieson DB, Loring DW (2004) Neuropsychological Assessment. New York, NY: Oxford University Press.

[24] Ivanoiu A, Adam S, Van der LM, Salmon E, Juillerat AC, et al (2005) Memory evaluation with a new cued recall test in patients with mild cognitive impairment and Alzheimer’s disease. J Neurol 252: 47–55. 15654553 10.1007/s00415-005-0597-2

[25] Wechsler D (1981) Wechsler Adult Intelligence Scale-Revised. New York, NY: Psychological Corporation.

[26] Delis DC, Kaplan E, Kramer JH (2001) Delis-Kaplan Executive Function System (D-KEFS) examiner’s manual. San Antonio, TX: The Psychological Corporation.

[27] Radloff L (1977) The CES-D Scale: A Self-Report Depression Scale for Research in the General Population. Appl Psychol Meas 1: 385–401.

[28] Rust J, Golombok S (2009) Modern Psychometrics, Second Edition: The Science of Psychological Assessment. Routledge, London & New York.

[29] Hvidtfeldt UA, Tolstrup JS, Jakobsen MU, Heitmann BL, Gronbaek M, et al. (2010) Alcohol intake and risk of coronary heart disease in younger, middle-aged, and older adults. Circulation 121: 1589–1597. CIRCULATIONAHA.109.887513 [pii];10.1161/CIRCULATIONAHA.109.887513 [doi]. 10.1161/CIRCULATIONAHA.109.887513 PMC3104851 20351238

[30] Cole SR, Hernan MA (2008) Constructing inverse probability weights for marginal structural models. Am J Epidemiol 168: 656–664. kwn164 [pii];10.1093/aje/kwn164 [doi]. 10.1093/aje/kwn164 PMC2732954 18682488

[31] Seaman SR, White IR (2011) Review of inverse probability weighting for dealing with missing data. Stat Methods Med Res. 0962280210395740 [pii];10.1177/0962280210395740 [doi]. 10.1177/0962280210395740 21220355

[32] Hernan MA, Robins JM (2006) Estimating causal effects from epidemiological data. J Epidemiol Community Health 60: 578–586. 60/7/578 [pii];10.1136/jech.2004.029496 [doi]. 10.1136/jech.2004.029496 PMC2652882 16790829

[33] Dufouil C, Ducimetiere P, Alperovitch A (1997) Sex differences in the association between alcohol consumption and cognitive performance. EVA Study Group. Epidemiology of Vascular Aging. Am J Epidemiol 146: 405–412. 9290500 10.1093/oxfordjournals.aje.a009293

[34] Arntzen KA, Schirmer H, Wilsgaard T, Mathiesen EB (2010) Moderate wine consumption is associated with better cognitive test results: a 7 year follow up of 5033 subjects in the Tromso Study. Acta Neurol Scand Suppl 23–29. ANE1371 [pii];10.1111/j.1600-0404.2010.01371.x [doi]. 10.1111/j.1600-0404.2010.01371.x 20586731

[35] Kalmijn S, van Boxtel MP, Verschuren MW, Jolles J, Launer LJ (2002) Cigarette smoking and alcohol consumption in relation to cognitive performance in middle age. Am J Epidemiol 156: 936–944. 12419766 10.1093/aje/kwf135

[36] Elias PK, Elias MF, D’Agostino RB, Silbershatz H, Wolf PA (1999) Alcohol consumption and cognitive performance in the Framingham Heart Study. Am J Epidemiol 150: 580–589. 10489997 10.1093/oxfordjournals.aje.a010056

[37] Lobo E, Dufouil C, Marcos G, Quetglas B, Saz P, et al. (2010) Is there an association between low-to-moderate alcohol consumption and risk of cognitive decline? Am J Epidemiol 172: 708–716. kwq187 [pii];10.1093/aje/kwq187 [doi]. 10.1093/aje/kwq187 20699263

[38] Tucker KL (2010) Dietary patterns, approaches, and multicultural perspective. Appl Physiol Nutr Metab 35: 211–218. h10-010 [pii];10.1139/H10-010 [doi]. 10.1139/H10-010 20383235

[39] Sabia S, Gueguen A, Berr C, Berkman L, Ankri J, et al. (2011) High alcohol consumption in middle-aged adults is associated with poorer cognitive performance only in the low socio-economic group. Results from the GAZEL cohort study. Addiction 106: 93–101. 10.1111/j.1360-0443.2010.03106.x [doi]. 10.1111/j.1360-0443.2010.03106.x PMC3006084 20840170

[40] Sinforiani E, Zucchella C, Pasotti C, Casoni F, Bini P, et al. (2011) The effects of alcohol on cognition in the elderly: from protection to neurodegeneration. Funct Neurol 26: 103–106. 4863 [pii]. PMC3814447 21729592

[41] Bate C, Williams A (2011) Ethanol protects cultured neurons against amyloid-beta and alpha-synuclein-induced synapse damage. Neuropharmacology 61: 1406–1412. S0028-3908(11)00363-7 [pii];10.1016/j.neuropharm.2011.08.030 [doi]. 10.1016/j.neuropharm.2011.08.030 21903110

[42] Collins MA, Neafsey EJ, Mukamal KJ, Gray MO, Parks DA, et al. (2009) Alcohol in moderation, cardioprotection, and neuroprotection: epidemiological considerations and mechanistic studies. Alcohol Clin Exp Res 33: 206–219. ACER828 [pii];10.1111/j.1530-0277.2008.00828.x [doi]. 10.1111/j.1530-0277.2008.00828.x PMC2908373 19032583

[43] Gross AL, Rebok GW, Ford DE, Chu AY, Gallo JJ, et al. (2011) Alcohol consumption and domain-specific cognitive function in older adults: longitudinal data from the Johns Hopkins Precursors Study. J Gerontol B Psychol Sci Soc Sci 66: 39–47. gbq062 [pii];10.1093/geronb/gbq062 [doi]. 10.1093/geronb/gbq062 PMC3001751 20937708

[44] Britton A, Singh-Manoux A, Marmot M (2004) Alcohol consumption and cognitive function in the Whitehall II Study. Am J Epidemiol 160: 240–247. 10.1093/aje/kwh206 [doi];160/3/240 [pii]. 10.1093/aje/kwh206 15257997

[45] Ganguli M, Vander BJ, Saxton JA, Shen C, Dodge HH (2005) Alcohol consumption and cognitive function in late life: a longitudinal community study. Neurology 65: 1210–1217. 65/8/1210 [pii];10.1212/01.wnl.0000180520.35181.24 [doi]. 10.1212/01.wnl.0000180520.35181.24 16247047

[46] Ngandu T, Helkala EL, Soininen H, Winblad B, Tuomilehto J, et al. (2007) Alcohol drinking and cognitive functions: findings from the Cardiovascular Risk Factors Aging and Dementia (CAIDE) Study. Dement Geriatr Cogn Disord 23: 140–149. 000097995 [pii];10.1159/000097995 [doi]. 10.1159/000097995 17170526

[47] Stampfer MJ, Kang JH, Chen J, Cherry R, Grodstein F (2005) Effects of moderate alcohol consumption on cognitive function in women. N Engl J Med 352: 245–253. 352/3/245 [pii];10.1056/NEJMoa041152 [doi]. 10.1056/NEJMoa041152 15659724

[48] Gordon R, Heim D, MacAskill S (2012) Rethinking drinking cultures: a review of drinking cultures and a reconstructed dimensional approach. Public Health 126: 3–11. S0033-3506(11)00267-8 [pii];10.1016/j.puhe.2011.09.014 [doi]. 10.1016/j.puhe.2011.09.014 22137093

